# Classification and prognostic factors of patients with cervical spondylotic myelopathy after surgical treatment: a cluster analysis

**DOI:** 10.1038/s41598-023-49477-4

**Published:** 2024-01-02

**Authors:** Xiao Fan, Rui Chen, Haoge Huang, Gangqiang Zhang, Shuai Zhou, Xin Chen, Yanbin Zhao, Yinze Diao, Shengfa Pan, Fengshan Zhang, Yu Sun, Feifei Zhou

**Affiliations:** 1 Department of Orthopaedics, Peking University Third Hospital, 49 North Garden Road, Haidian District, Beijing, 100191 China; 2 Engineering Research Center of Bone and Joint Precision Medicine, 49 North Garden Road, Haidian District, Beijing, 100191 China; 3 Beijing Key Laboratory of Spinal Disease Research, 49 North Garden Road, Haidian District, Beijing, 100191 China

**Keywords:** Prognosis, Risk factors, Skeleton, Orthopaedics

## Abstract

Identifying potential prognostic factors of CSM patients could improve doctors’ clinical decision-making ability. The study retrospectively collected the baseline data of population characteristics, clinical symptoms, physical examination, neurological function and quality of life scores of patients with CSM based on the clinical big data research platform. The modified Japanese Orthopedic Association (mJOA) score and SF-36 score from the short-term follow-up data were entered into the cluster analysis to characterize postoperative residual symptoms and quality of life. Four clusters were yielded representing different patterns of residual symptoms and quality of patients’ life. Patients in cluster 2 (mJOA RR 55.8%) and cluster 4 (mJOA RR 55.8%) were substantially improved and had better quality of life. The influencing factors for the better prognosis of patients in cluster 2 were young age (50.1 ± 11.8), low incidence of disabling claudication (5.0%) and pathological signs (63.0%), and good preoperative SF36-physiological function score (73.1 ± 24.0) and mJOA socre (13.7 ± 2.8); and in cluster 4 the main influencing factor was low incidence of neck and shoulder pain (11.7%). We preliminarily verified the reliability of the clustering results with the long-term follow-up data and identified the preoperative features that were helpful to predict the prognosis of the patients. This study provided reference and research basis for further study with a larger sample data, extracting more patient features, selecting more follow-up nodes, and improving clustering algorithm.

## Introduction

Cervical spondylotic myelopathy (CSM), as a common senile degenerative disease, has become a worldwide health problem, bringing serious burdens to individuals and society^1^. The pathogenesis of CSM is thought to be related to spinal cord compression, but the exact pathophysiological mechanism remains unclear^2^. Currently, the main treatment for CSM is spinal cord decompression by surgery, but the efficacy of surgery varies with individual differences^3,4^. Preoperatively, the severity of the disease is mainly evaluated based on the patient's symptoms and imaging parameters, but this pattern still has certain limitations in predicting the patient's curative effect^5^. Previous studies have shown that preoperative factors such as age, severity of spinal cord compromise, duration of symptoms, comorbidities and cervical sagittal alignment can affect the prognosis of patients^6–10^. Nevertheless, in most of these studies patient classification is based on research purposes and clinical experience, leading to limitation of patient characteristics, patient-based assessment such as the quality of life and inconsideration of confounding factors.

As an exploratory data analysis method, cluster analysis refers to the analysis process of grouping a collection of samples into multiple categories composed of similar samples, so that the samples have a high degree of intra-group similarity and inter-group difference. In this process, cluster analysis can automatically classify from sample data without relying on the classification criteria given by researchers in advance. Therefore, from the perspective of machine learning, cluster analysis is an unsupervised learning process. Cluster analysis based on unsupervised machine learning can improve the classification of disease phenotypes and patients in studies^11,12^, and with incorporating more clinical features, previously unobvious data associations and structures may be revealed^13^. In this study, it is assumed that there are clinically related groups in existing CSM patients that transcend the previous prior classification, and hierarchical clustering is applied to explore the types of patients, and the types of patients generated by the clustering are analyzed, so as to identify the preoperative related factors with predictive significance for the mixed system, and to determine which patients have the best surgical effect.

## Data and methods

### General information

Based on the clinical big data research platform of our institution, data of CSM patients who received surgical treatment in our hospital from January 2012 to December 2020 were collected. Inclusion criteria: (1) CSM patients diagnosed by orthopedics and undergoing surgical treatment (anterior cervical discectomy and fusion [ACDF] or laminoplasty [LP]); (2) Age > 18 years old; (3) Complete preoperative baseline data and follow-up data including at least one short-term (≤ 6 months) and one long-term (≥ 12 months) follow-up. Exclusion criteria: (1) previous history of neck surgery; (2) unqualified follow-up time; (3) patients with traumatic myelopathy or cervical spine deformities.

### Baseline data

1. *Population characteristics*: gender, age, smoking and alcohol consumption history.
2. *Clinical symptoms*: numbness, neck and shoulder pain, chest and abdominal banding sensation, plantal cotton-stepping sensation, fine motor loss, gait abnormality, sympathetic symptoms;
3. *Physical examination*: muscular atrophy, muscle strength loss, abnormal reflexes, positive pathological signs (included Rossolimo’s sign, Hoffmann’s sign or Babinski’s sign), positive Eaton test, positive Spurling test;
4. *Scoring Information*: modified Japanese Orthopedic Association score (mJOA)^14^ and Quality of Life Short Form 36 (SF-36) scale^15^ were used to assess cervical spinal cord function and preoperative quality of life respectively.

In the above data, gender is a binary variable, while age and score data are continuous variables. Smoking history, alcohol consumption history, clinical symptoms and physical examination were all defined as "yes/no" binary variables.

### Follow-up data

Short-term follow-up data within 6 months and long-term follow-up data over 12 months after surgery were included in this study. If patients had multiple follow-ups during this period, the follow-up timepoint farthest from the operation time was selected respectively. The follow-up included mJOA score and SF-36 scale, in which the data of SF-36 scale included scores of eight dimensions including physical function (PF), role-physical (RP), bodily pain (BP), vitality (VT), social function (SF), role-emotional (RE), mental health (MH) and general health (GH), and SF-36 health transformation (HT) score.

### Patient prognosis assessment

In this study, the spinal cord Recovery Ratio (RR) was used to evaluate the prognosis of patients. Spinal cord function RR = (follow-up mJOA score − baseline mJOA score)/(17-baseline mJOA score) × 100%^16^. An improvement rate of > 50% was defined as a good prognosis^17^.

### Statistical analysis

SPSS (IBM, 24.0) was used for statistical analysis of the data.

1. In this study, hierarchical clustering was selected as the clustering method, and the clustering characteristic values included mJOA and SF-36 scores of short-term follow-up patients. The square of Euclidean distance was selected to measure the similarity of objects, and Ward's method was used for clustering calculation^18^. According to the dendrogram generated by hierarchical clustering and the elbow graph drawn by sum of the squared errors (SSE) and clustering number k, the optimal clustering number was finally selected.
2. For patients in each group determined by hierarchical clustering, the spinal cord function improvement rate (RR) based on mJOA score was calculated to show the prognosis of patients in each group, and the prognosis of patients in each group during long-term follow-up was analyzed to test the effectiveness of cluster analysis. And finally the impact of baseline data on the prognosis of patients in each group was analyzed.
3. For continuous variables such as age and score data, they were expressed as mean ± standard deviation; Shapiro–Wilk test was used to evaluate whether the data were normally distributed. For the data with normal distribution, one-way ANOVA was used to test the differences between the groups. For data with non-normal distribution, the rank sum test (Kruskal–Wallis test) was used to analyze the differences between groups. For the other classification variables, the expected frequency was calculated first. For the data with expected frequency ≥ 5, Pearson Chi-square test (χ^2^) was used to analyze the differences among all groups. For data with expected frequency < 5, Fisher's exact test was used to analyze the differences among groups.

### Ethical approval

This study was approved by the Ethics Committee of Peking University Third Hospital (2021-60-02). Informed consent was obtained from all subjects in the database. All analysis was performed in accordance with relevant regulations of the committee and the Declaration of Helsinki.

## Results

**Figure 1:**
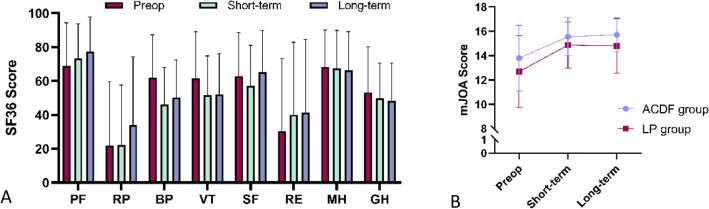
Baseline data and follow-up data of mJOA score and SF-36 score for the entire cohort. The change of each SF-36 score is different, and the SF-36 data of each patient is quite different. (**A**) The mJOA score shows a trend of improvement in both ACDF group and LP group (**B**).

In this study, 476 patients with CSM (249 males and 227 females) with an average age of 52.0 ± 11.2 years were included, including 71 patients (14.9%) with a history of smoking and 40 patients (8.4%) with a history of alcohol consumption. Clinical symptoms: 389 patients (81.7%) with numbing, 106 patients (22.3%) with neck and shoulder pain, 28 patients (5.9%) with chest and abdominal band sensation, 185 patients (38.9%) with plantar cotton-stepping sensation, 59 patients (12.4%) with fine motor loss, 40 patients (8.4%) with gait abnormality, 96 patients (20.2%) with sympathetic symptoms; Physical examination: 49 patients (10.3%) with muscle dystrophy, 255 patients (53.6%) with decreased muscle strength, 43 patients (9.0%) with abnormal reflex, 337 patients (70.8%) with positive pathological signs, 183 patients (38.4%) with positive Eaton test, 92 patients (19.3%) with positive Spurling test. Among all patients, 355 (74.6%) had surgery involving multilevel of the cervical spine. 288 (60.5%) patients underwent ACDF, and 188 (39.5%) underwent laminoplasty (LP). There was no significant difference in prognosis of patients between ACDF and LP group, with a short-term mJOA RR (%) 43.3 ± 60.8 vs 41.6 ± 51.3, P = 0.078, and a long-term mJOA RR (%) 44.0 ± 79.5 vs 39.1 ± 68.5, P = 0.073. Baseline and follow-up data of mJOA score and SF-36 score for the entire cohort are shown in Table 1 and Fig. 1.

**Table 1:** Baseline data and follow-up data of mJOA score and SF-36 score for the entire cohort.

Variables	Score		
	Baseline	Follow-up	
		Short-term	Long-term
SF-36 PF	68.9 ± 25.5	73.3 ± 20.5	77.4 ± 20.4
SF-36 RP	21.8 ± 37.7	22.1 ± 35.5	34.0 ± 40.2
SF-36 BP	62.0 ± 25.3	46.1 ± 22.0	50.3 ± 22.1
SF-36 VT	61.6 ± 27.5	51.7 ± 23.1	52.1 ± 23.9
SF-36 SF	62.9 ± 25.6	57.0 ± 24.0	65.3 ± 24.5
SF-36 RE	30.2 ± 43.0	40.0 ± 42.9	41.4 ± 43.2
SF-36 MH	68.4 ± 21.8	67.5 ± 22.4	66.3 ± 22.8
SF-36 HT	4.4 ± 3.2	3.2 ± 1.3	3.2 ± 1.2
SF-36 GH	53.3 ± 26.8	49.7 ± 20.8	48.4 ± 22.2
mJOA score	13.4 ± 2.8	15.3 ± 1.7	15.4 ± 1.8

### Results of hierarchical cluster analysis

**Figure 2:**
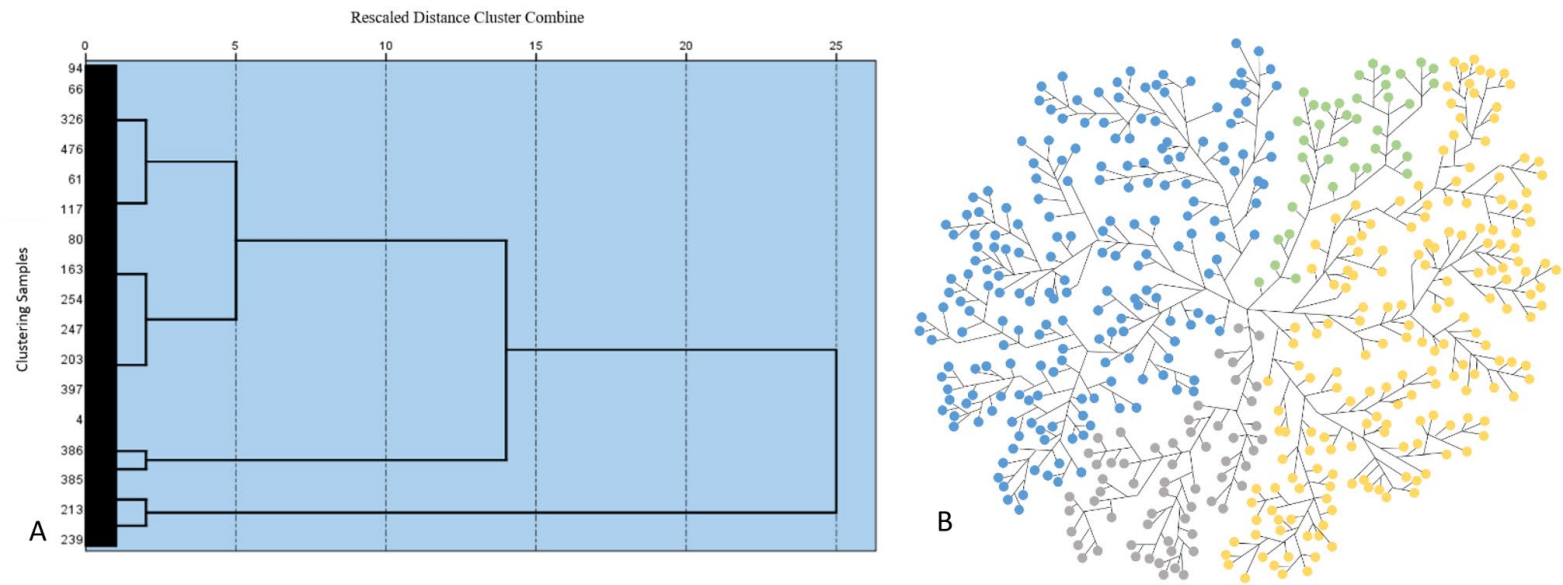
Dendrogram from application of unsupervised hierarchical clustering. It shows 2 definite clusters at the first branch, 3 clusters at the second branch and 4 clusters at the third branch. (**A**) Each dot represents a patient of the sample and colors represent the portions of the dendrogram in each of the four patient clusters. Green represents cluster 1, yellow cluster 2, blue cluster 3 and gray cluster 4.

**Figure 3:**
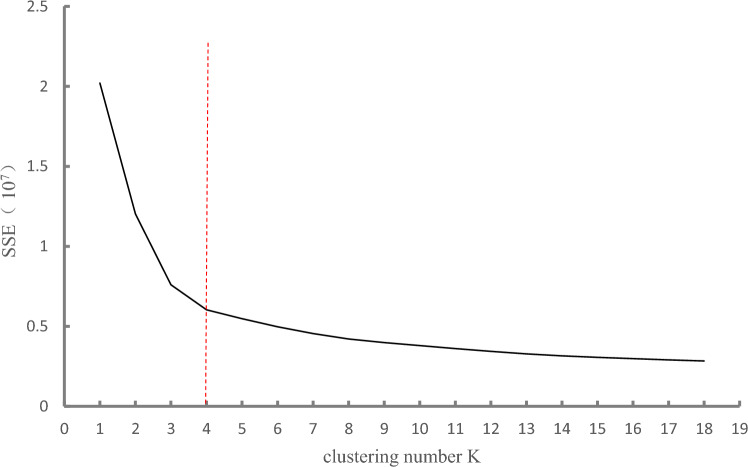
“Elbow” relation between the sum of the squared errors (SSE) and clustering number k. SSE is the clustering error of all samples. With the increase of clustering number k, the aggregation degree of each category will gradually increase, and SSE will become smaller. When k is less than the real cluster number, the increase of k value will greatly increase the aggregation degree of each category, and the corresponding SSE decreases greatly. However, when k reaches the real clustering number, the aggregation degree improved by increasing k becomes smaller rapidly, so SSE tends to get flat with the continuous increase of k value. Therefore, the graph shape of SSE and k resembles "elbow", and the value of k corresponding to the inflection point of the elbow was 4.

The results of hierarchical clustering of 476 patients are shown in the dendrogram (Fig. 2). Refer to the relation between the sum of squared errors (SSE) and clustering number k (Fig. 3), the optimal clustering number in this study was 4. Table 2 shows the total sample of CSM patients included in this study and the score data of the 4 clusters of patients generated by hierarchical clustering. Among them, the short-term follow-up score was the features included in hierarchical cluster, with significant difference among all clusters (P < 0.001). Preoperative score data of all clusters showed significant differences in SF-36 physiological function (P = 0.008). There were significant differences in mJOA scores (P = 0.042), and no significant differences in other preoperative scores (P > 0.05, Table 2).

**Table 2:** Score data of patients in each cluster.

Variables	Results after hierarchical clustering				P value^a^
	Cluster 1	Cluster 2	Cluster 3	Cluster 4	
Subjects (%)	38 (8.0%)	181 (38.0%)	197 (41.4%)	60 (12.6%)	
Preoperative scores					
SF-36 PF	71.1 ± 23.5	73.1 ± 24.0	65.4 ± 25.7	66.1 ± 28.5	P = 0.008
SF-36 RP	19.1 ± 36.5	26.0 ± 39.9	19.7 ± 36.4	17.9 ± 35.7	P = 0.175
SF-36 BP	62.8 ± 28.7	62.8 ± 24.9	60.8 ± 25.0	63.3 ± 25.3	P = 0.628
SF3-6 VT	57.5 ± 30.3	62.9 ± 26.0	61.2 ± 28.7	61.1 ± 26.8	P = 0.797
SF-36 SF	64.5 ± 24.9	66.0 ± 23.9	59.8 ± 26.5	62.1 ± 27.4	P = 0.122
SF-36 RE	23.6 ± 39.4	36.7 ± 44.7	27.5 ± 42.4	23.4 ± 40.3	P = 0.056
SF-36 MH	66.3 ± 24.8	69.7 ± 20.9	67.6 ± 22.3	68.1 ± 21.3	P = 0.853
SF-36 HT	4.3 ± 0.7	4.5 ± 4.9	4.4 ± 1.3	4.4 ± 0.7	P = 0.111
SF-36 GH	57.9 ± 28.3	54.9 ± 25.5	52.3 ± 27.6	48.6 ± 26.7	P = 0.258
mJOA score	13.6 ± 2.2	13.7 ± 2.8	13.1 ± 2.9	13.0 ± 2.9	P = 0.042
Follow-up scores					
SF-36 PF	76.2 ± 20.6	79.9 ± 17.9	65.9 ± 21.4	76.2 ± 16.7	P < 0.001
SF-36 RP	6.9 ± 10.1	33.8 ± 40.1	14.1 ± 31.0	22.5 ± 34.4	P < 0.001
SF-36 BP	54.0 ± 20.6	53.9 ± 20.5	37.3 ± 20.8	46.5 ± 20.4	P < 0.001
SF3-6 VT	59.5 ± 25.9	58.7 ± 21.3	42.4 ± 21.2	56.2 ± 22.5	P < 0.001
SF-36 SF	66.8 ± 23.5	65.6 ± 23.0	46.8 ± 21.4	58.3 ± 22.9	P < 0.001
SF-36 RE	13.6 ± 11.7	54.9 ± 43.6	30.4 ± 41.0	43.2 ± 44.0	P < 0.001
SF-36 MH	78.8 ± 21.0	75.5 ± 19.5	57.7 ± 20.9	68.6 ± 23.1	P < 0.001
SF-36 HT	2.6 ± 1.2	3.5 ± 1.2	3.0 ± 1.3	3.3 ± 1.3	P < 0.001
SF-36 GH	50.7 ± 22.6	55.8 ± 20.0	44.4 ± 20.0	48.4 ± 19.8	P < 0.001
mJOA score	15.2 ± 1.9	15.7 ± 1.4	14.8 ± 1.8	15.4 ± 1.7	P < 0.001

^a^Using the rank sum test (Kruskal–Wallis test) to analyze the difference between groups, and P < 0.05 represented a significant difference.

**Figure 4:**
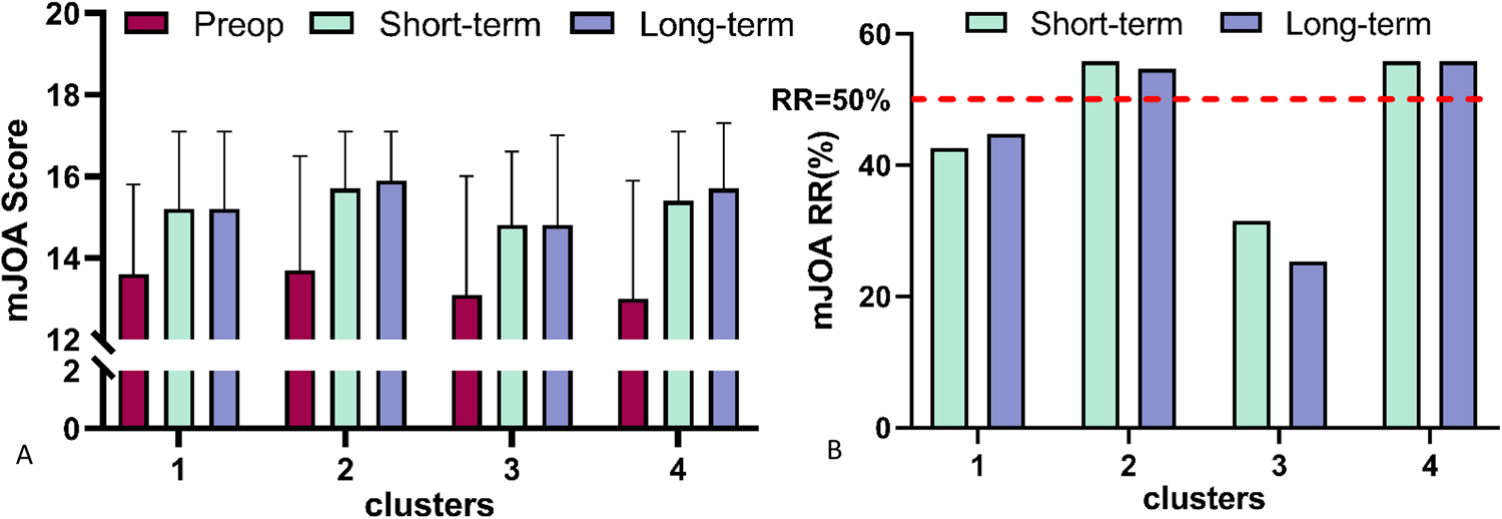
mJOA score and RR of patients in each cluster. The mJOA scores of patients in each cluster were improved postoperatively. (**A**) The recovery ratio of mJOA score (mJOA RR) was used to evaluate the prognosis of patients in each cluster. (**B**) The mJOA RR of patients in cluster 2 and 4 were more than 50%, indicating a good prognosis, while those in cluster 1 and 3 had a poor prognosis.

The prognosis of each cluster was mainly reflected by the improvement rate of mJOA score after surgery. Cluster 2 and 4 had higher mean mJOA improvement rates, while cluster 1 and 3 had lower mean mJOA improvement rates, with significant differences among clusters. (P < 0.001, Table 3) Meanwhile, patients in each cluster showed similar results during long-term follow-up: the mJOA RR in cluster 2 and 4 was significantly higher than that in cluster 1 and 3 (Fig. 4).

**Table 3:** mJOA score and RR of patients in each cluster.

Variables	Cluster 1	Cluster 2	Cluster 3	Cluster 4	P value
Short term follow-up					
mJOA score	15.2 ± 1.9	15.7 ± 1.4	14.8 ± 1.8	15.4 ± 1.7	P < 0.001
mJOA RR (%)	42.6 ± 50.6	55.8 ± 50.8	31.5 ± 62.4	55.8 ± 57.1	P < 0.001
Long term follow-up					
mJOA score	15.2 ± 1.9	15.9 ± 1.2	14.8 ± 2.2	15.7 ± 1.6	P < 0.001
mJOA RR (%)	44.7 ± 52.7	54.6 ± 73.5	25.3 ± 80.6	55.8 ± 57.1	P < 0.001

Table 4 shows the differences in the distribution of CSM patient population characteristics, clinical symptoms, physical examination and surgical procedures among all clusters. The population difference among all clusters was manifested in age (P = 0.021). Preoperative clinical symptoms were different in neck and shoulder pain (P = 0.044) and gait abnormality (P = 0.012). The difference of preoperative physical examination was in the proportion of patients with positive pathological signs among all clusters (P = 0.006). There was no significant difference in other characteristics of patients (P > 0.05, Table 4).

**Table 4:** Population characteristics, clinical symptoms and physical examination features of patients in 4 clusters.

Variables	Results after hierarchical clustering				P value
	Cluster 1	Cluster 2	Cluster 3	Cluster 4	
Subjects (%)	38 (8.0%)	181 (38.0%)	197 (41.4%)	60 (12.6%)	
Age (years)	55.0 ± 10.5	50.1 ± 11.8	52.9 ± 10.0	52.7 ± 12.6	P = 0.021
Gender (M/F)	21/17	101/80	98/99	29/31	P = 0.590
Smoking history (y/n)	7/31	28/153	30/167	6/54	P = 0.666
Alcohol consumption history (y/n)	4/34	15/166	16/181	5/55	P = 0.970
Numbness (y/n)	33/5	147/34	161/36	49/11	P = 0.866
Neck and shoulder pain (y/n)	7/31	51/130	41/156	7/53	P = 0.044
Chest and abdominal banding sensation^a^ (y/n)	3/35	8/173	14/183	3/57	P = 0.609
Cotton-stepping sensation (y/n)	15/23	64/117	79/118	27/33	P = 0.568
Fine motor loss^a^ (y/n)	3/35	22/159	25/172	9/51	P = 0.791
Gait abnormality^a^ (y/n)	8/30	9/172	16/181	7/53	P = 0.012
Sympathetic symptoms (y/n)	5/33	34/147	39/158	18/42	P = 0.172
Muscular atrophy^a^ (y/n)	3/35	20/161	20/177	6/54	P = 0.985
Muscle strength loss (y/n)	21/17	91/90	106/91	37/23	P = 0.490
Abnormal reflexes^a^ (y/n)	2/36	19/162	15/182	7/53	P = 0.569
Positive pathological signs (y/n)	30/8	114/67	142/55	51/9	P = 0.006
Positive Eaton test (y/n)	11/27	54/127	48/149	14/46	P = 0.593
positive Spurling test (y/n)	5/33	46/135	31/166	10/50	P = 0.068
Surgical procedure (ACDF/LP)	24/14	123/58	108/89	33/27	P = 0.052

Age was a continuous variable and had a normal distribution by Shapiro–Wilk test. One-way ANOVA was used to test the difference between groups.

^a^These features had an expected frequency < 5, so that Fisher's exact test was used to analyze the differences among groups. For other features with expected frequency ≥ 5, Pearson Chi-square test (χ^2^) was used to analyze the differences among all groups.

## Discussion

### Advantages of cluster analysis

Cluster analysis is an unsupervised machine learning method that can find more homogeneous groups in different data sets^19^. This approach can identify more complex data patterns in more realistic hybrid systems and classify patients or interventions based on observable eigenvalues. In cluster analysis, determining patient types and intervention categories are purely data-driven that does not rely on a priori assumptions and is therefore an effective complement to supervised learning.

Hierarchical clustering, as one of the methods of cluster analysis, does not need to specify the number of clusters in advance, and can show the clustering process of large samples directly. Ames, etc.^13^ proposed that hierarchical clustering based on artificial intelligence can be used to include and synchronously analyze more overall patient population characteristics, symptom factors, imaging and functional scores than existing patient classification schemes. Similarly, the method has been used to describe groups of patients in various diseases, including adult spinal deformities, pulmonary hypertension, asthma, mental disorders, and malignancies^20–23^, and the influencing factors of patient benefit after surgical intervention^24^.

### Significance of CSM patient categories obtained based on hierarchical clustering method

With the baseline data of the patients included in the study, this study reviewed the literature on CSM in recent years, summarized the common symptoms and signs, and combined with the data of our hospital to screen the specialty characteristics of cervical spondylosis with high frequency, included in this study. The mJOA score scale is the most used objective index to evaluate the outcome of patients with CSM^14^. But previous studies^10,25^ showed a lack of patient-based assessment such as the quality of life. In addition to spinal cord function, the improvement of patients' health-related quality of life more directly demonstrates the therapeutic effect from the perspective of patients. Therefore, the results of SF-36 scale were also included in the selection of clustering features in this study.

In this study, long-term follow-up data of the same population were analyzed to preliminarily verify the rationality of the hierarchical clustering results based on short-term follow-up data. A previous 10-year follow-up study^26^ found that mJOA scores showed similar improvement rates within one year or more for patients after CSM surgery, and this trend continued until 5 years after surgery. In this study, the mJOA RR in each cluster during long-term follow-up was consistent with that in short-term follow-up, indicating that the hierarchical clustering results based on short-term follow-up data were representative to a certain extent. Thus, it is reasonable to use cluster 2 and 4 to represent patients with good prognosis in this study.

From the results of hierarchical clustering, surgical intervention can significantly improve spinal cord function in CSM patients, which is consistent with the results of a systematic review by Rhee et al.^27^ in 2017. Affected by preoperative factors, the prognosis of the four clusters of patients was different.

**Figure 5:**
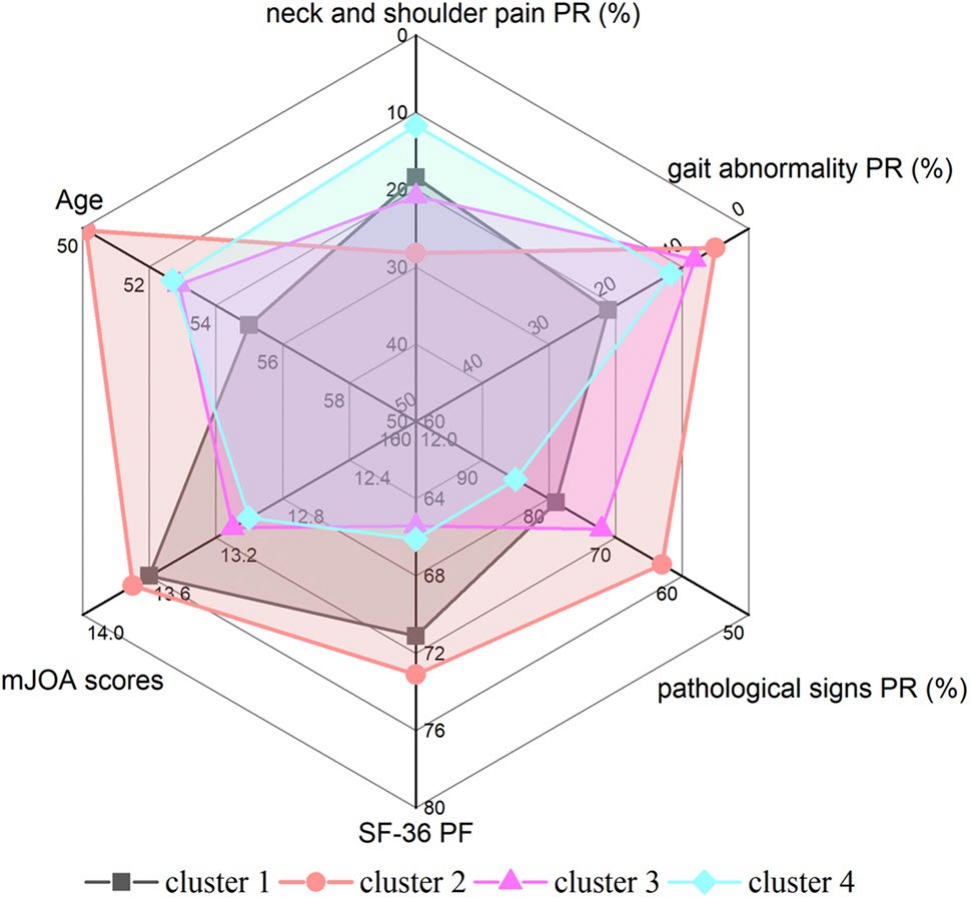
Radar map of prognostic factors of patients in each clusters. The proportion of color on the map represents the prognosis of patients in each cluster. *PR* positive ratio, *PF* physical function.

In terms of population characteristics, the average age of patients was younger in the cluster 2 and 4 with a good prognosis, while the average age of patients in the cluster 1 and 3 with a poor prognosis corresponded to a larger average age (Fig. 5). Matsuda et al. conducted a study on 17 CSM patients over 70 years old and reported that their recovery rate was significantly lower than that of the control group^28^. In a prospective study, Furlan et al.^29^ reported similar results, and this study suggests that such age-related prognostic differences are also present in younger age groups. However, Hasegawa et al.^30^ and Holly et al.^31^ reported that compared with younger CSM patients, there is no significant difference in surgical outcomes in older patients, but the incidence of neurological complications is higher. This may be contributed to the fact that (1) the spinal cord in the elderly experiences age-related changes, including decreased C-motor neurons, decreased number of anterior horn cells, and decreased number of myelinated fibers in the corticospinal tract and posterior cord^32^; (2) older patients are more likely to have unrelated comorbidities that may affect quality of life^33^. Kusin et al.^34^ reported that smoking is also an important prognostic factor in CSM patients, but there was no similar trend found in our cluster analysis. The reasons may be that (1) most patients choose to quit smoking before admission; (2) the personal history of medical history was not collected carefully, resulting in inaccurate baseline data, and the impact of local air pollution on non-smokers.

In terms of clinical symptom and physical examination, compared with patients in the cluster 2 and 3, it can be found that lower rate of abnormal gait and positive rate of pathological signs before surgery also correspond to better prognosis of patients, similar to the specific symptoms and signs mentioned by Badhiwala et al.^5^ which may also be potential predictors of outcome in patients with CSM, but the biological mechanism remained unclear. Previous studies^25,35^ reported that gait abnormality was a disturbing symptom in patients with CSM, which can result from the involvement of long tracts of the spinal cord.

In terms of preoperative score data, it is reported in the systematic review by Tetreault et al.^33^ that the duration of symptoms and the severity of preoperative myelopathy are also clear predictors of prognosis in CSM patients. The results of this study suggest that preoperative myelopathy severity can be better reflected by SF-36 physical function (PF) score and mJOA score, which are significantly higher in cluter 2 with a good prognosis than in the other groups, while the cluster 3 with a poor prognosis corresponds to a poor preoperative score. In addition, by comparing patients in cluster 3 and 4, it could be speculated that age and incidence of neck and shoulder pain may be influencing factors to determine the prognosis of patients with poor preoperative SF-36 physical function (PF) score and mJOA score. Whether they may be useful clinical factors for identifying CSM patients at risk for poor postoperative outcomes deserves further studies.

In the field of CSM, Zhou et al.^36^ previously proved that machine learning-based clustering could be used to rationally classify a heterogeneous cohort of CSM patients effectively. In this study, through the postoperative cluster analysis of CSM patients and combined with the baseline data of patients, the preoperative population characteristics and clinical characteristics affecting the prognosis of patients were screened out. In future studies, the amount of information of patients will be larger, and the influence of various factors on the surgical prognosis of patients will be more complex and diversified. Unlike traditional research methods such as cohort study, cluster analysis can identify more valuable preoperative related factors in a hybrid system closer to the real world, and make more accurate preoperative curative effect prediction based on these factors.

### Limitations

In this study, patients were unsupervised divided into 4 categories and some possible prognostic factors for CSM patients were screened out, which is helpful to provide useful information for informed consent of patients before surgery and assist doctors in clinical decision-making^18^. However, this study also has shortcomings: (1) As a retrospective study, the data in this study was restricted in a single center, and complete data of patients only accounted for 25.1% with a relatively serious situation of data missing and loss of follow-up. Still, this is already a relatively large cohort based on a review of similar studies of cervical spine disease^37^. (2) This study did not include preoperative and follow-up imaging data for analysis, and did not collect patients' previous history data (e.g., diabetes), detailed surgical data (e.g., segments involved, blood loss) and other postoperative rehabilitation data, which may have a certain impact on our patient clustering^38^. (3) Compared with the analysis of multiple perioperative time points, the duration of this study was fairly short, which may have limitations in describing the duration of patients' symptoms and recovery course^24^. (4) Blind evaluation is recommended for clinical studies; however, mJOA scores are evaluated by surgeons. The data from this process will likely affect the results of the experiment^18^.

In conclusion, the postoperative efficacy of CSM patients related to this study still needs to be verified by multi-center, large sample size and long-term follow-up observation. Despite the above limitations, the results of this study preliminarily verified the feasibility of hierarchical clustering in the study of prognostic factors of CSM patients, laying a foundation for future cluster studies involving surgical information, imaging information and other factors.

## Conclusions

In this study, cluster analysis was performed based on postoperative follow-up information, and CSM patients who underwent surgical treatment were divided into four categories, representing four different prognostic patterns of patients, from which preoperative factors were identified and could help predict the prognosis of patients: (1) lower age in the population characteristics; (2) lower rates of neck and shoulder pain and gait abnormalities among clinical symptoms; (3) a smaller positive rate of pathological signs on physic al examination; (4) higher SF-36 physiological functional dimension scores and mJOA scores in the scoring information, all of above referred to better patient outcomes. This study explored the feasibility of applying cluster analysis method in the study of prognostic factors of CSM patients, and provided reference and research basis for further relevant studies, which may include collecting larger sample data, extracting more patient characteristics, setting more follow-up timepoints, and improving the clustering algorithm.

## Data Availability

The data used in this study are available in the Hospital Data Respository Console of Peking University Third Hospital, but restrictions apply to public availability of these data used under license for the current study. Reasonable request for access to the database could be made by contacting the corresponding author for detailed process.
